# Seasonal Changes in a Maize-Based Polyculture of Central Mexico Reshape the Co-occurrence Networks of Soil Bacterial Communities

**DOI:** 10.3389/fmicb.2017.02478

**Published:** 2017-12-18

**Authors:** Eria A. Rebollar, Edson Sandoval-Castellanos, Kyria Roessler, Brandon S. Gaut, Luis D. Alcaraz, Mariana Benítez, Ana E. Escalante

**Affiliations:** ^1^Departamento de Ecología Evolutiva, Instituto de Ecología, Universidad Nacional Autónoma de México, Mexico City, Mexico; ^2^Centro de Ciencias de la Complejidad, Universidad Nacional Autónoma de México, Mexico City, Mexico; ^3^Department of Ecology and Evolutionary Biology, University of California, Irvine, Irvine, CA, United States; ^4^Laboratorio Nacional de Ciencias de la Sostenibilidad, Instituto de Ecología, Universidad Nacional Autónoma de México, Mexico City, Mexico; ^5^Facultad de Ciencias, Universidad Nacional Autónoma de México, Mexico City, Mexico

**Keywords:** milpa, bacterial diversity, co-occurrence networks, seasonal agriculture, Actinobacteria, Proteobacteria, Chloroflexi

## Abstract

The milpa is a traditional maize-based polyculture in Mexico that is typically practiced as rainfed agriculture. Because milpa cultivation has been practiced over a vast range of environmental and cultural conditions, this agroecosystem is recognized as an important repository of biological and cultural diversity. As for any agroecosystem, the relationship between plant development and the biogeochemical processes of the soil is critical. Although the milpa has been studied from different perspectives, the diversity and structure of microbial communities within milpa soils remain largely unexplored. In this study, we surveyed a milpa system in Central Mexico across cropping season: before planting (dry season; *t1*), during the early growth of plants (onset of the rainy season; *t2*), and before harvest (end of the rainy season; *t3*). In order to examine changes in community structure through time, we characterized bacterial diversity through high-throughput sequencing of 16S rRNA gene amplicons and recorded the nutrient status of multiple (5–10) soil samples from our milpa plots. We estimated microbial diversity from a total of 90 samples and constructed co-occurrence networks. Although we did not find significant changes in diversity or composition of bacterial communities across time, we identified significant rearrangements in their co-occurrence network structure. We found particularly drastic changes between the first and second time points. Co-occurrence analyses showed that the bacterial community changed from a less structured network at (*t1*) into modules with a non-random composition of taxonomic groups at (*t2*). We conclude that changes in bacterial communities undetected by standard diversity analyses can become evident when performing co-occurrence network analyses. We also postulate possible functional associations among keystone groups suggested by biogeochemical processes. This study represents the first contribution on soil microbial diversity of a maize-based polyculture and shows its dynamic nature in short-term scales.

## Introduction

Soil microbes play a primary role in ecosystem functions and sustainability, including agricultural ecosystems (Wardle et al., 2004; van der Heijden et al., 2008). In agroecosystems, productivity, resilience to perturbations, nutrient cycling, and resistance to plagues is strongly influenced by soil microbial biodiversity (Van Bruggen and Semenov, 2000). Microbial communities change their composition and function as a consequence of environmental changes and farming practices (Fierer et al., 2007; Strickland et al., 2009; Lage et al., 2010); however, there is still little understanding about the nature and relative contribution of the specific factors that affect the composition and structure of soil microbial communities in time and space (Barberán et al., 2011; Wood et al., 2015; Shi et al., 2016).

In recent years, the composition and structure of microbial communities has been reported in many ecosystems; many studies on this topic have been published thanks to the development of high-throughput sequencing technologies (Metzker, 2010; Caporaso et al., 2011) and the use of analytic methods such as co-occurrence networks (Barberán et al., 2011). The use of these methods has helped identify some of the factors that contribute to soil microbial diversity and structure within agroecosystems (Shi et al., 2016). In studies with maize and rice, for example, large effects on microbial diversity are associated with soil type and cultivation practices (Peiffer et al., 2013; Edwards et al., 2015). However, bacterial diversity surveys for agricultural soils have focused mainly on the characterization of microbial communities assessed in a single time-point and mostly on crop monocultures. Crop polycultures, however, are very important because of their central role in the development of sustainable agriculture (Perfecto et al., 2009; Chappell et al., 2013). Moreover, they are often subjected to drastic environmental and management changes throughout the year, while being highly dependent on rainwater. For example, nearly three quarters of the agricultural production in rural Mexico is rainfed (SAGARPA, 2014). Given seasonal variation in rainfall, studies of polycultures should include longitudinal sampling that captures potential seasonal changes.

The milpa is a traditional polyculture in Mexico and Mesoamerica that is based on maize and has been recognized as an invaluable repository of biological and cultural diversity (Altieri, 2004; García-Barrios et al., 2009; Chappell et al., 2013). The milpa typically includes intercropping of maize and common beans but often features additional crops such as tomato, squash, chili, jicama, and avocado. Over thousands of years, this polyculture has been adapted to a variety of climatic, edaphic, and cultural conditions, and it has been the foundation of food security in many Latin American rural communities (Altieri et al., 2012). The milpa system has been studied from different perspectives. Some of the bacterial diversity associated with milpa soils has been characterized but only for particular microbial species and families (Silva et al., 2003, 2005). Nevertheless, to our knowledge, no studies have been conducted on the structure and diversity of milpa-associated bacterial communities. Taking into account the recognized values of the milpa, it is of interest to investigate its associated microbiota, particularly for the conservation or restoration of the microorganism-mediated biogeochemical processes that can be the base of an input-free and sustainable agriculture (Chappell et al., 2013; FAO, 2015).

**FIGURE 1:**
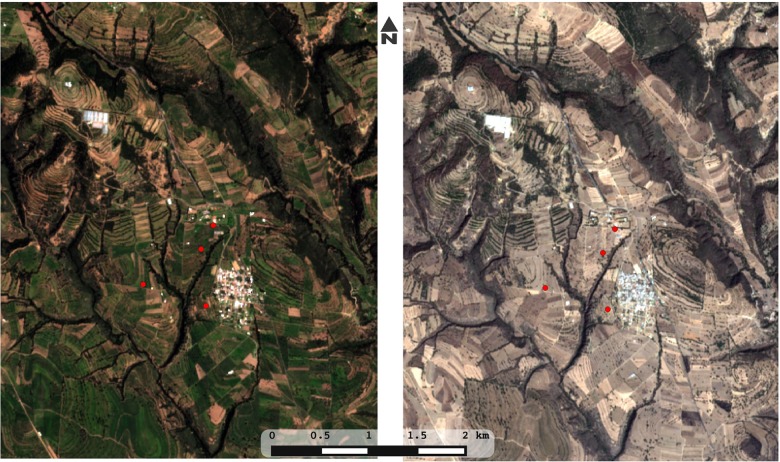
Plots of rain-fed milpa Tlaxcala, Mexico. Four plots of maize-based polyculture were sampled in three key time points (before planting/dry season, early growth of plants/beginning of the rain season, before harvest/end of the rain season). Within each plot, five points were sampled with replicates, adding to 90 soil and DNA samples for the whole study. Images were obtained in true color from the satellite Sentinel-2 covering the Españita municipality in Tlaxcala, Mexico. In order to illustrate the drastic seasonality of the site, an image from the 2016 rainy season (left) is compared with an image from the 2017 dry season (right).

In the present study, we report the composition and structure of soil prokaryotic communities associated with milpa plots in the central highlands of Mexico, in a region where small farmers practice rain-fed maize agriculture with several plants in association or in rotation (**Figure 1**). Given the marked seasonality of milpa agriculture in this region we explore not only the composition and structure of soil prokaryotic communities but also their seasonal change along the cropping season. We hypothesize that nutrient profiles, bacterial diversity, bacterial composition, and co-occurrence networks exhibit seasonal changes. For testing this hypothesis, we have collected soil samples from four plots at three key time points in the agricultural cycle. We determined the pH and the total content of nitrogen, carbon and phosphorus, and characterized the microbial community by means of high-throughput 16S rRNA amplicon sequencing. Finally, we interpreted the correlations among microbial taxa in terms of their ecological roles and putative interactions (Dunne et al., 2002; Montoya and Solé, 2002).

## Materials and Methods

### Study Site

The four milpa plots for this study (F, L, T, and R) are located in the Españita municipality, in the state of Tlaxcala in Mexico (around 19°07′08″N 98°10′12″W; **Figure 1**). Since 1997, This municipality has been influenced by the “Proyecto de Desarrollo Rural Integral Vicente Guerrero”, a small rural farming organization that practices and promotes agroecological strategies (Holt-Jimenez, 2008). Agriculture in this community is performed by small farmers in plots that range from 0.5 to 2 Ha. We investigated the management history of each plot through informal interviews with the farmers and with the organization representatives, and found that all plots cultivate a diversity of plants besides maize (beans, squash, tomato, etc.), usually in association but sometimes in a rotation scheme. Considering this, the chosen plots were a reasonable representation of the milpa grown throughout the central Mexican highlands.

### Sampling

We sampled four milpa plots for bulk soil (**Figure 1**). In all cases, we sampled at three time points: (i) before planting (dry season; *t1*), (ii) during the early growth of plants (onset of the rainy season; *t2*), and (iii) before harvest (end of the rainy season; *t3*). The first time-point was done in May, the second in July and the third in September, all in 2013. For each plot and time point we sampled 5 to 10 plot-replicates in a longitudinal transect: detailed samples sizes used for community analysis (90 in total) and nutrient composition (60 in total) are shown in Supplementary Table S1. The difference in the total number of samples we analyzed between physicochemical and community analyses is due to the fact that in *t2* and *t3* we collected two samples per site only for the community analyses obtaining a total of 100 samples. However, 10 of these samples did not retrieve optimal sequencing results so we ended up with a total of 90 samples (see Supplementary Table S1 for details). In *t2* and *t3*, plants were already growing, thus the two samples per sites corresponded to 5 and 20 cm distance from the plants. After analysis, the distance from the plant did not explain any differences in microbial diversity or composition; thus, these samples were considered as duplicates.

For the genomic procedures, we collected approximately 30 g of surface soil; for *t2* and *t3* we marked plants and sampled the same spot. All samples were immediately frozen in liquid nitrogen in the field and transported to the laboratory for further procedures. For soil physicochemical parameters analysis (60 samples in total), we collected 500 g of bulk soil for the same sampling points described above.

### Laboratory Procedures

#### DNA Extraction

Soil samples were sieved through a 2 mm soil mesh to remove small branches, leaves and rocks. Genomic DNA was extracted using PowerSoil DNA Isolate Kit^TM^ (MoBio Laboratories, Solana Beach CA, United States), with a slightly modified protocol (0.25 g of sample, all 4°C incubation times increased to 20 min, and addition of a 55°C incubation step prior to DNA elution).

#### Amplification and Sequencing

The 16S rRNA gene was amplified with the 515F/806R primers that target the V4 region (Caporaso et al., 2011). PCR amplifications were performed in a total volume of 25 μl and included 1 μl of template DNA, along with 0.2 μM of each PCR primer. PCR conditions followed those of Caporaso et al. (2011). Individual PCR products were quantified on a Qubit fluorometer (Singapore) and combined into a multiplex, which was purified on Qiaquick columns (Qiagen, Valencia, CA, United States). The eluted multiplex was then size-fractionated on a low temperature 1% agarose gel; a band of the expected size of ∼300 bp was extracted, and the band was purified using QIAQuick Gel Extraction Kit^TM^ (Qiagen from Qiagen, Valencia, CA, United States). The pooled sample was sequenced on an Illumina HiSeq2500, using 250 bp paired-end reads of 150 cycles.

#### Soil Physicochemical Parameters Determination

Soil pH was measured with a digital pH meter (Corning), using deionized water (1:2 w/v). Previous to nutrient determination, a 100 g aliquot of soil was oven-dried at 75°C to constant weight. Total C was determined by dry combustion and coulometric determination (Huffman, 1977) using a Total Carbon Analyzer (UIC Mod. CM5012; Chicago, United States). Total N and P in soil were extracted by acid digestion with H_2_SO_4_, H_2_O_2_, K_2_SO_4_, and CuSO_4_ at 360°C. Total N concentration was determined using a modified Kjeldahl method (Bremmer, 1996) and P concentration was determined by colorimetry, using the molybdate-ascorbic acid method (Murphy and Riley, 1962). Both were quantified with a Bran-Luebbe Auto Analyzer III (Norderstedt, Germany).

### Nutrient Contents Data Analysis

#### Statistical Analyses

All statistical analysis for the nutrient data were conducted in R (R Core Team, 2014) using the vegan package (Oksanen et al., 2016). Given the nested nature of our sample scheme (samples from different plots in different sampling times), we conducted a non-parametric nested analysis of variance based on 1000 permutations (PERMANOVA using adonis function), and a *post hoc* Wilcoxon test, in order to distinguish differences in nutrient content associated with the sample origin (plot) and sampling time. All the scripts developed in R are available online at: https://github.com/LANCIS-escalante-lab/milpa.

### 16S rDNA Sequences Analyses

#### De-multiplexing, Filtering, and Chimera Check

Illumina raw sequences were processed with Quantitative Insights Into Microbial Ecology pipeline, QIIME (Caporaso et al., 2010a). First, sequences were de-multiplexed, using local scripts. Next, paired-end reads were joined into contigs using join_paired_ends.py with default arguments. Joined sequences were filtered for quality based on two criteria: (i) sequences with more than 2 N’s were removed and (ii) sequences with overall (average) phred quality scores <20 were discarded. These steps removed 48.4% of the total number of reads, leaving 32,965,400 total reads, an average of 343,389.6 for each of the samples. The presence of chimeras was checked with Chimeraslayer (Haas et al., 2011). Chimeric sequences (1.7% of the total reads) were eliminated and the rest of the sequences were filtered by size keeping only the sequences with 228–230 bp in length.

The raw data (paired end files) were deposited in the NCBI sequence read archive (SRA) with the accession numbers SRR5957113 (Biosample SAMN07501976) for R1 files and SRR5942330 (Biosample SAMN07501975) for R2 files. Both files can be found as part of the Bioproject PRJNA398138.

#### OTU Assignment

De-multiplexed and filtered sequences (30,138,961 of reads) were clustered into operational taxonomic units (OTUs) at a sequence similarity threshold of 97% with the UCLUST method (Edgar, 2010). Sequences were matched against the Greengenes database (May 2013 release; McDonald et al., 2012), and those that did not match the database were clustered as *de novo*. Taxonomy was assigned using the RDP classifier (Wang et al., 2007) and the Greengenes database. Representative sequences were aligned to the Greengenes database with PyNAST (Caporaso et al., 2010b), and a ML phylogenetic tree was constructed with FastTree 2 (Price et al., 2010). The obtained OTU table was filtered using a minimum cluster size of 0.001% of the total number of reads, i.e., we kept OTUs with more than 300 reads (Bokulich et al., 2013).

### Statistical Analyses of Molecular Data

#### Diversity and Statistical Analyses

To evaluate differences across times and plots, the Shannon diversity index was calculated, and ANOVA and *post hoc* Tukey tests were conducted using R (R Core Team, 2014). To evaluate if the sample size had an effect on Shannon diversity per time, we performed 1000 random subsamplings of the data set so that *t1*, *t2*, and *t3* had equal sample size (17 samples). We performed 1000 ANOVAs and identified the proportion of *P* > 0.05. To calculate beta diversity, we obtained a Weighted Unifrac distance matrix and distances were visualized with a Principal Coordinates Analysis (PCoA). Differences in beta diversity across time were tested with a two factor non-parametric analysis of variance based on 999 permutations (PERMANOVA) using the software PRIMER-E (Clarke and Gorley, 2006). To evaluate if the sample size had an effect on beta diversity per time, we resampled the Weighted Unifrac matrix (1000 times) so that *t1*, *t2*, and *t3* had equal sample size (17 samples). We performed 1000 PERMANOVAs and identified the proportion of *P* > 0.05. All alpha and beta diversity metrics, PCoAs, and relative abundance descriptions of the soil communities at the phylum level were obtained with QIIME (Caporaso et al., 2010a). Sample based subsampling trials to test the effect of sample size on alpha and beta diversity were done in R (R Core Team, 2014).

#### Network Inference Analysis

The networks were constructed with the software CoNet v1.1.0 (Faust and Raes, 2016) by using tables of OTUs abundances at the family level obtained with QIIME (see above). We constructed one network with all the samples pooled together, and separate networks among the different plots (F, L, T, and R) and among the different time points (*t1*, *t2*, and *t3*).

We set a minimum of occurrences among replicates to 20–25% and normalized the values. The co-occurrences were tested statistically with Pearson, Spearman, and Kendall tests as well as with the dissimilarity index of Bray–Curtis. For all tests, only correlations >0.5 (and Bray–Curtis distances <0.5) and with *P* < 0.05 were considered as significant. Edges were established when the co-occurrences/exclusions were supported by at least three out of the four (correlations/dissimilarity) indices. The values of the edges corresponded to the average value among indexes. We also applied a multi-test correction with both a Fisher’s *Z* and the Benjamini–Hochberg procedure (Benjamini and Hochberg, 1995), with *q*-values set to 0.05.

To describe the structure of the inferred networks we calculated standard network indexes, tested a fit of the distribution of connectivity to a power law, and calculated modules. The calculation of network indexes as well as the visualization and manipulation of networks were all carried out in the software Cytoscape v3.3.0 (Cline et al., 2007), which also assisted the construction of networks by running CoNet. To test if the taxa appeared randomly distributed among modules, we applied a contingency table test with the frequencies of taxa inside modules. We assessed the re-allocation of nodes in modules by computing the ratios of nodes that persisted in modules between *t1* and *t2*, and between *t2* and *t3*, and visualized those changes by means of alluvial diagrams, constructed with the MapEquation online engine (Rosvall and Bergstrom, 2010). Further, we investigated if modules displayed not only temporal changes in their nodes composition but also changes in their internal structure. For that goal, we compared the sets of nodes’ pairwise distances between consecutive time-points. The distance between nodes that we employed was the length of the shortest path between nodes pairs, which was the sum of absolute edges’ weights subtracted from one (recall that edges weights were average correlation values). We obtained the distances patterns for both modules and taxa, and compared those patterns between successive time-points by means of a correlation coefficient *R*^2^.

We assessed the consistency of modules by computing the entire network modularity with four methods: the Greedy Modularity Optimization, Short Random Walks, Matrix Eigenvector, and Simulated Annealing.

The pipeline we followed has several measures for improving robustness, including some that would weaken edges established by statistical artifacts associated to small or uneven sampling. In particular, from all the measures available in CoNet, the three tests and the distances that we used are not reported among those that are particularly sensitive to sample number (Faust and Raes, 2016). However, since the number of samples is different between *t1* and *t2*/*t3*, we re-built networks for each time-point taking a random downsample for times *t2* and *t3*. For this exercise, we performed a double randomization step as a measure to improve robustness due to small sampling following Faust and Raes (2016).

## Results

### Soil Physicochemical Parameters Show Differences in Time

Results from the nutrient analysis are presented in **Table 1**. From these data, we investigated the variation in nutrient contents among plots and across time points through a non-parametric nested analysis of variance (PERMANOVA). The results showed significant differences in nutrient content among sampling times (time; *F* = 2.6412, *P* = 0.035). In addition, plot explained differences in nutrient content (plot; *F* = 11.4215, *P* = 0.000999), but we did not detect a significant interaction between plot and time (interaction *F* = 1.9281, *P* = 0.06939). *Post hoc* Wilcoxon tests identified differences across time points: we found a significant difference between *t2* and the other two sampling times due to pH (**Table 1**). Finally, we found that *t1* and *t3* differ in the C:P ratio (Supplementary Table S2).

**Table 1:** Soil physicochemical parameters.

Variable

	pH			C (mg⋅g^-1^)			N (mg⋅g^-1^)			P(mg⋅g^-1^)		
				
Sampling time	*t1*	*t2*	*t3*	*t1*	*t2*	*t3*	*t1*	*t2*	*t3*	*t1*	*t2*	*t3*
R-plot	6.73 ± 0.31	5.37 ± 0.38	6.72 ± 0.84	6.53 ± 1.18	4.20 ± 0.40	6.32 ± 2.36	0.45 ± 0.13	0.37 ± 0.06	0.48 ± 0.13	0.18 ± 0.05	0.33 ± 0.32	0.28 ± 0.08
L-plot	6.38 ± 0.05	5.82 ± 0.24	5.6 ± 0.07	6.05 ± 0.97	6.64 ± 0.81	6.46 ± 1.06	0.45 ± 0.06	0.36 ± 0.09	0.42 ± 0.08	0.28 ± 0.05	0.32 ± 0.13	0.38 ± 0.05
T-plot	6.80 ± 0.40	5.88 ± 0.11	6.50 ± 0.32	24.34 ± 5.29	12.36 ± 0.90	8.20 ± 0.93	1.14 ± 0.34	0.78 ± 0.05	0.58 ± 0.06	0.48 ± 0.19	0.40 ± 0.00	0.36 ± 0.06
F-plot	6.52 ± 0.44	6.32 ± 0.36	6.60 ± 0.17	9.60 ± 1.50	8.32 ± 0.87	13.87 ± 4.77	0.68 ± 0.15	0.60 ± 0.07	0.77 ± 0.12	0.38 ± 0.11	0.42 ± 0.05	0.37 ± 0.06


*Mean ± standard deviation of the four studied milpa plots (R, L, T, F) and the three sampling times (*t1*, *t2*, *t3*).*

### Bacterial Diversity and Community Structure Do Not Exhibit Seasonal Changes

**FIGURE 2:**
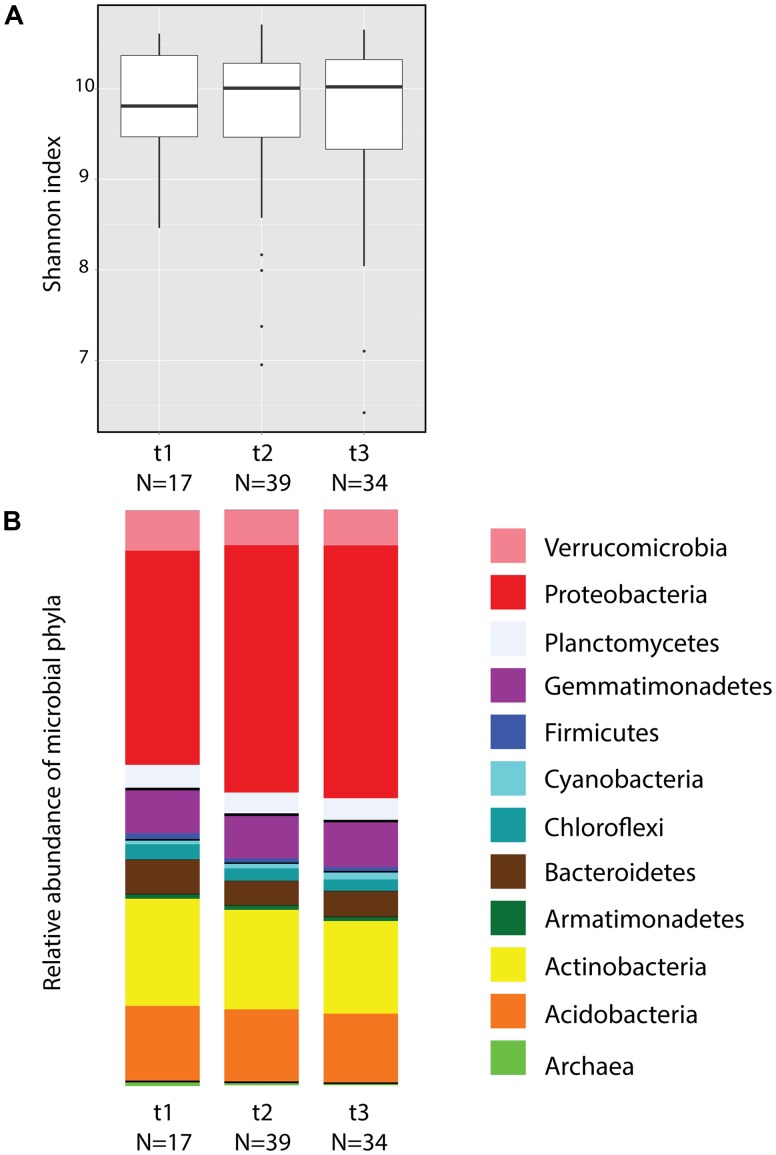
Soil microbial community diversity across three time points of pooled samples. **(A)** Box plot of alpha diversity estimates (Shannon index) obtained from soil microbial communities. **(B)** Mean relative abundance of the 12 most abundant bacterial and archaeal phyla.

A total of 7,183 OTUs were identified from the 90 soil samples collected during three time points (after filtering to 119,062 reads the total number of reads for the collection of samples was 10,715,580). Considering all time points, the five dominant phyla were Proteobacteria (41.35%), Actinobacteria (17.33%), Acidobacteria (12.47%), Gemmatimonadetes (7.53%), and Verrucomicrobia (6.41%) (**Figure 2B**). According to Shannon index estimates, no significant differences in alpha diversity were found across time (**Figure 2A**, ANOVA) or among plots with the exception of plot R, which differed from plots L and T only at *t2* (Supplementary Figure S1; ANOVA *F*_(3,35)_ = 3.747, *P* = 0.0196). Random subsamplings of the data set to balance sample size, showed that 99.5% of the time the effect of Time was not significant (ANOVA *P* > 0.05). Principal coordinate analyses (Supplementary Figure S2) showed no significant differences across time according to beta diversity estimates using Weighted Unifrac distances (Adonis test: Pseudo-*F*_(2,87)_ = 1.4254, *P* = 0.102). However random subsampling of the Weighted Unifrac distance matrix indicated that, when sample size per time is equal (17 samples), the effect of time was significant 21.3% of the time (PERMANOVA *P* < 0.05).

### Co-occurrence Patterns Show Significant Changes in Time

We obtained co-occurrence networks for the four plots, the three time-points, and the combination of both categories.

The networks obtained for the three time points (*t1*, *t2*, and *t3*) fitted a Power law (*R*^2^ = 0.75–0.81) and displayed strong modularity and hierarchical properties (see below), all of which have been associated with network complexity (Ravasz and Barabási, 2003; Barabási and Oltvai, 2004). Moreover, a significant Power law fit was also observed in sub-networks that constitute taxonomic groups even when lower taxonomic hierarchies were used, or when taxa were taken inside modules.

The comparisons between plots, which were made by pooling together the three time-points, showed no relevant differences in size and network indexes (Supplementary Table S3). In agreement with the network indexes, these networks looked similar and compact (Supplementary Figure S4). Networks inferred for each combination of plot and time-point retained some complexity properties as a power law distribution of degree and modularity. However, they were too small and did not displayed remarkable differences (see Supplementary File S1).

**FIGURE 3:**
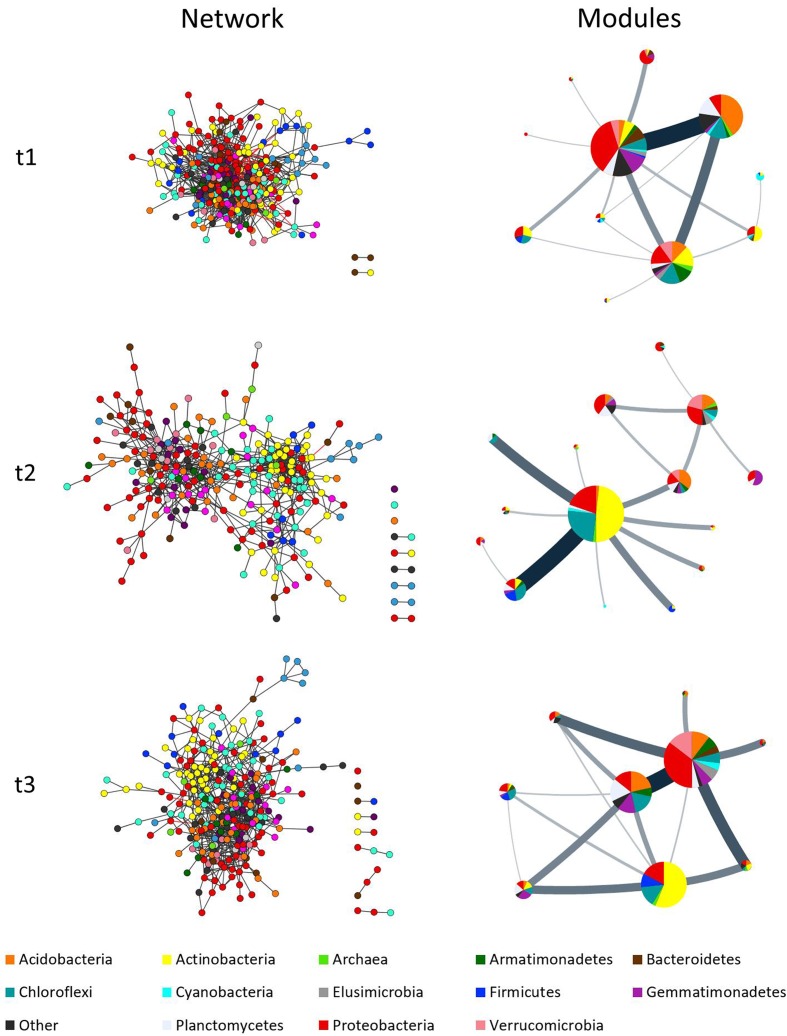
Complex co-occurrence networks of microbial communities of milpa soil. Networks correspond to three time points: *t1* = before planting (dry season); *t2* = during the early growth of plants (onset of the rainy season); and *t3* = before harvest (end of the rainy season). Charts at left show the original networks with nodes colored by taxa while charts at right show the condensed networks where each circle represent a module with their size being equivalent to the size of the module (nr. of nodes), and the taxa share displayed as a pie chart. Line thickness indicates amount of “flow” (edges) between modules. Network inference was done considering diversity at family level.

The comparison between time-points showed statistically relevant differences in their size, network indexes, and modularity (**Table 2** and **Figure 3**). The differences were larger between *t1* and *t2* than between *t2* and *t3*, in good agreement with the analyses of soil physicochemical parameters in which *t1* and *t2* showed larger differences than *t2* and *t3* (**Table 1** and Supplementary Table S3). For instance, the *t1* and *t2* networks shared 205 edges (which represent 12 and 31% of *t1* and *t2* networks, respectively) while *t2* and *t3* shared 252 edges (which represent 38 and 30% of *t2* and *t3* networks, respectively), and *t3* and *t1* shared 244 edges (representing 35 and 17%, respectively). The *t1* network was more densely connected (*d* = 0.038) than *t2* and *t3* (*d* = 0.018 and 0.021, respectively; **Table 2** and **Figure 3**). The overall differences between networks at different time-points were maintained after performing the robustness test suggested by Faust and Raes (2016) (data not shown).

**Table 2:** Network indices for three time points: (*t1*), before planting (dry season); (*t2*) during the early growth of plants (onset of the rainy season), and (*t3*) before harvest (end of the rainy season).

	Sampling time		
	
Network index	*t1*	*t2*	*t3*
Number of nodes	302	266	267
Number of edges	1685	650	825
Connectivity	11.159 ± 9.210	7.0695 ± 7.954	8.192 ± 8.165
Clustering coefficient	0.210 ± 0.166	0.149 ± 0.198	0.157 ± 0.208
Betweenness centrality	0.007 ± 0.008	0.015 ± 0.020	0.022 ± 0.099
Closeness centrality	0.327 ± 0.094	0.273 ± 0.161	0.309 ± 0.146
Average shortest path length	3.213 ± 0.686	4.105 ± 1.199	3.555 ± 1.037
Network density	0.037	0.018	0.023
Network heterogeneity	0.824	0.826	0.817
Network centralization	0.106	0.057	0.067
Power law of node degree, *R*^2^	0.752	0.809	0.799


*When applicable, ± values correspond to standard deviation. Network inference was done considering diversity at the family taxonomic level.*

**FIGURE 4:**
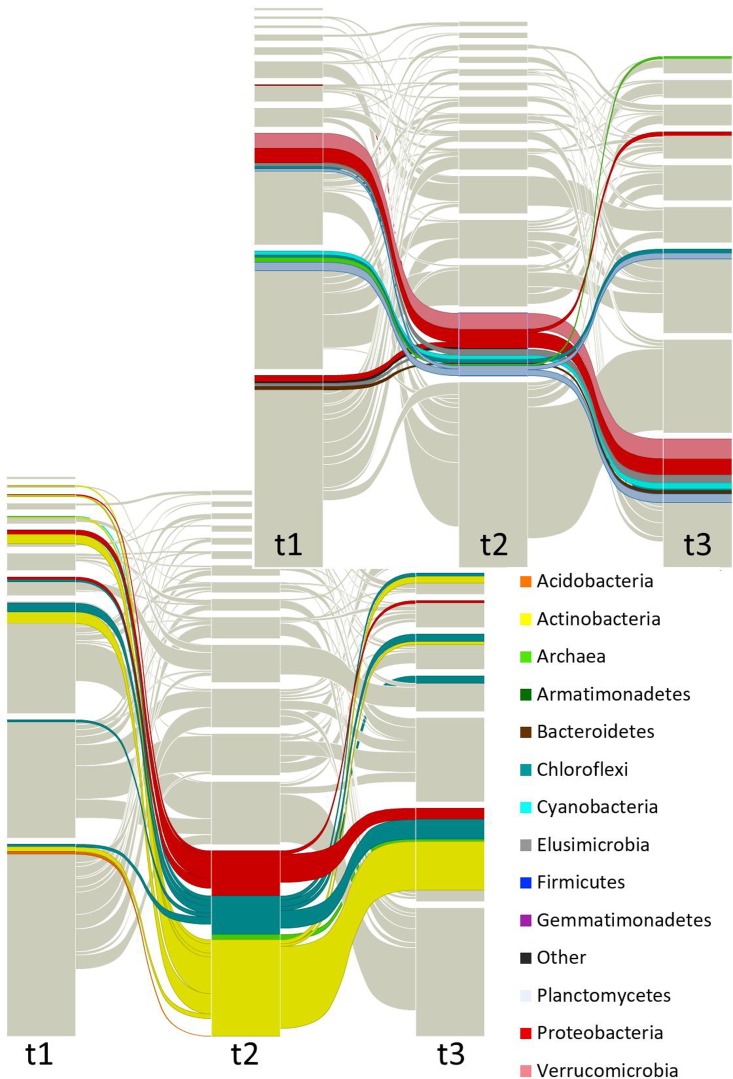
Focused alluvial diagram of three times. Each column represents a time (*t1* = before planting (dry season); *t2* = during the early growth of plants (onset of the rainy season); *t3* = before harvest (end of the rainy season)) and the blocks at each time the network modules. The flow lines among times represent the module re-assignation of groups of OTUs (nodes). Colors correspond to taxa as indicated in the list, but only the two largest modules (at *t*2) were colored to avoid saturation of the figure. The top graph is highlighting one dominant module and the bottom graph is showing another dominant module.

**Figures 3**, **4** and Supplementary Figure S3 show that taxa re-allocation in modules occurred extensively between time-points *t1* and *t2* and moderately between *t2* and *t3*, especially for three phyla: Actinobacteria, Chloroflexi and Proteobacteria. The proportional representation of taxa in modules was non-random (*t1*: χ^2^ = 263, d.f. = 56, *P* < 0.001; *t2*: χ^2^ = 165, d.f. = 48, *P* < 0.001; *t3*: χ^2^ = 158, d.f. = 56, *P* < 0.001). As for the modules persistence, the three largest modules of *t1* had a low persistence in *t2* (11.9, 27.3, and 25%) but the five largest modules of *t2* were highly persistent in *t3* (55.4, 75, 35.3, 20, and 63.2%; **Figure 4** and Supplementary Figure S3). The structure of pairwise distances of modules followed a similar pattern: low persistence between *t1* and *t2* (*R*^2^ = 0.2 to 0.47, mean = 0.28) and noticeably a higher persistence between *t2* and *t3* (*R*^2^ = 0.18 to 0.62, mean = 0.47) (Supplementary Figure S5). When we compared the sets of pairwise distances of taxa in the different time-points, we found the same tendency of being more similar between *t2* and *t3* than between *t1* and *t2* (*R*^2^ = 0.04 to 0.69, mean = 0.35 for *t1*–*t2* and *R*^2^ = 0.42 to 0.83, mean = 0.63 for *t2*–*t3*) (Supplementary Figure S6).

## Discussion

### Seasonal Changes Are Associated with Changes in Physicochemical Soil Parameters

Management of soil in the milpa agriculture is tightly associated with rain and its accompanying environmental changes. Due to the rainfed nature of the milpa agricultural system, moisture can be considered one of the key environmental parameters, which has been reported (for other soil study systems) as an influential variable affecting bacterial community structure as well as carbon and nitrogen transformations (Fierer and Schimel, 2002; Fierer et al., 2003). Seasonal changes in the milpa system, as in other agroecosystems, also include anthropogenic changes associated with cropping including tillage and fertilization, which have also been associated with changes in microbial communities (Yin et al., 2010). Finally, temperature, humidity, and microbial activity are additional potential drivers of ecosystem changes.

In the present study, we characterized some of the potential outputs of seasonality and cultivation practices on soil properties. Although we do not have specifics about the inputs (e.g., tillage type, fertilization) associated with management, we did document the practices of plot owners through informal communication, particularly with respect to management strategies associated with preparing the land for planting (between *t1* and *t2*). Among the physicochemical variables measured from the studied soil samples, pH best explains the differences among sampling time points, specifically acidification in *t2*. This may be caused by the application of fertilizer inputs, which were applied prior to planting and hence prior to *t2*. Previous studies have shown that the physicochemical reactions that take place after the fertilizer application reduce pH by enhancing proton release nitrification and ammonium uptake by the plants (Francioli et al., 2016). Acidification of soil in turn can lead to nutrient depletion (Barack et al., 1997), affecting microbial biomass (Lupwayi et al., 2011; Lazcano et al., 2013) and enzyme activities (Nannipieri and Gianfreda, 1998; Guo et al., 2011; Gianfreda and Ruggiero, 2006). In fact, soil pH has been widely accepted as a critical factor impacting composition and diversity of soil bacterial communities (Fierer and Jackson, 2006; Lauber et al., 2009; Zhalnina et al., 2014), and recent evidence shows that different types of fertilization can affect soil microbial communities in maize agroecosystems (Zhang et al., 2017). Regardless of the specific physicochemical or microbial changes throughout the cropping season, our observations show the importance of a short-term temporal perspective of agroecosystems.

### Seasonality Is Not Reflected in Microbial Diversity

Despite the fact that we found significant changes in at least two physicochemical variables among sampling times, no significant changes were observed in alpha and beta diversity across time points. Even though diversity estimates are useful to describe communities, these are not always informative about the consequences of different treatments/conditions, as typified by this study where no statistically significant differences were observed across time points. However, we were able to distinguish trends in which the relative abundance of certain taxa (e.g., Proteobacteria, Actinobacteria), change slightly from *t1* to *t2–t3* (**Figure 2**). In addition, beta-diversity analysis shows a similar situation, in which *t1* samples correspond to a slightly different ordination than *t2–t3* (Supplementary Figure S2). As indicated by previous studies (Anderson and Walsh, 2013), beta diversity estimates can be influenced by unbalanced sample sizes. Considering the latter, in this study we identified a significant effect of time (21.3% of the time) when we performed subsamplings of the matrix equalizing the sample size per time. These results suggest that the trend observed in Supplementary Figure S2 could be obscured by different sample sizes on each time point (17, 39, 34).

The lack of significant differences in diversity are in contrast with previous studies in which significant differences in diversity can be found across both time and space (e.g., Buckley and Schmidt, 2003). In this context, we presume either that the temporal scale for the sample is inappropriate to document changes in microbial diversity or that the system (rain fed agriculture) is more resilient to environmental changes. Further studies that include sampling in more than one agricultural cycle will be needed to better understand the mechanisms involved in these patterns.

### Co-occurrence Networks Reveal Other Aspects of the Microbial Diversity That Can Inform Further Studies

The inferred co-occurrence networks exhibit a power law distribution, a high degree of modularity, and a hierarchical nature. These properties have been found in other biological networks and have been associated with complexity and robustness (e.g., Albert et al., 2000; Melián and Bascompte, 2002; Bastolla et al., 2009). From these network properties, modularity has been proposed to reflect habitat heterogeneity, divergent selection or phylogenetic clustering of related species, generating nonrandom patterns of association (Pimm and Lawton, 1980; Lewinsohn et al., 2006; Olesen et al., 2007). In this study, we observed a taxonomical enrichment of modules and found that the power law, a property of complex networks not necessarily present in random sub-sets of our networks, was maintained in subsets defined by taxonomical groups. This suggests that some complex network properties are brought about by the ecological relationships inside and among taxa and call for future studies analyzing the phylogenetic component of the networks.

Significant changes in co-occurrence networks were found across time points. In contrast to the standard diversity and composition analyses, the analyses of networks detected large changes between *t1* and *t2*, including a full scale re-arrangement of modules, a change in the pattern of distances among nodes of the entire network, and the redistribution of taxa in modules. One of the interesting aspects of these co-occurrence networks, is that some of the main phyla in these communities (Proteobacteria, Actinobacteria, and Chloroflexi) rearrange across time points. These co-occurrence patterns are of interest if we think about them from the perspective of functional ecology, particularly since the grouping of these phyla happens just after the onset of the rainy season, when plants start to grow and fertilizers have been added. While these patterns were robust to a randomization test and were obtained from a pipeline that minimizes error associated to small or uneven sampling (Methods), it is in principle possible that the differences in the number of samples at *t1* and *t2*/*t3* introduce artifacts in the comparison among *t1*, *t2*, and *t3* networks. Further studies are needed to fully assess the potential effect of small or uneven sampling in the modularity of module composition of co-occurrence networks.

Previous studies looking at the ecological roles of phyla in soil have identified Proteobacteria and Firmicutes as copiotrophs or fast growing organisms that prefer carbon-rich environments that satisfy their high demands of energy to maintain their growth rates (Fierer et al., 2007). In contrast, groups such as Chloroflexi have been reported to be very slow growers (Davis et al., 2011) that may rely on whatever minimal resources are available. Finally, members of the Actinobacteria, one of the predominant phyla in this study, have been reported to play an important role as organic matter decomposers (Strap, 2011), which may be of key importance in maintaining microbially mediated processes when nutrients become limited after fertilization and plant uptake. Given this, we could speculate on the cooperative behavior of these groups; where at the face of nutrient depletion, Chloroflexi, as a slow grower phyla (Davis et al., 2011) can thrive given the slow demand of nutrients, while Actinobacteria act as the decomposers that release the nutrients required by the fast-growers such as Proteobacteria. This persistence of co-occurring taxa, with some relative abundance fluctuations (i.e., Actinobacteria, Chloroflexi, and Proteobacteria) among time-points suggests that the persistence of these modules in *t2*–*t3* could represent ecologically meaningful assemblages, something that has been reported in similar, more controlled, maize-vegetable rotation systems (Zhang et al., 2017). In particular, Zhang et al. (2017) reported that Proteobacteria are always present in agricultural soils, with little or no fluctuations in time or in response to agricultural practices that alter some physicochemical properties (i.e., pH), but groups such as Actinobacteria or Chloroflexi, despite being present, showed contrasting patterns of relative abundance in response to fertilization and consequent pH changes. In this regard, it is tempting to think that the network modules represent microbial assemblages that play specific functions in the soil ecosystem of the milpa. Further studies, looking specifically at agricultural practices and temporal changes in relative abundance and co-occurrence patterns of functional groups and genes are needed to investigate these hypotheses.

## Conclusion

Given the vast diversity and functional redundancy of microorganisms, it remains unclear which factors control specific changes and, to some extent, whether microbial community structure actually matters for ecosystem functioning (Allison and Martiny, 2008). In this work, we assessed short-term temporal changes of bacterial communities in the milpa agroecosystem and found, by employing diverse experimental and analytical techniques, that these communities are robust in their composition and structure in the spatial scale, but that they change in their overall organization over the short-term. These temporal changes coincide with seasonal differences, plant growth, and the addition of fertilizers, which are followed by physicochemical changes in the soil. In the context of the current biodiversity and food crisis (Chappell et al., 2013), it has become crucial to address the study of agroecosystems and food production from an interdisciplinary perspective. In this scheme, the study of microbial communities across time and space is fundamental to understand nutrient cycling and the role of climate, especially on rain-fed and diverse agroecosystems like the milpa.

## Author Contributions

ER: Conducted all the diversity analyses for the sequence data, constructed the corresponding figures and wrote the manuscript. ES-C: Conducted all the network analyses from the co-occurrence database, constructed the corresponding figures and wrote the manuscript. KR: Filtered and organized the raw sequences files. BG: Conceived and designed the research, collected samples, revised all versions of the manuscript. LA: Conceived and designed the research, collected samples, revised final version of the manuscript. MB: Conceived, designed and conducted the research, directed the network analyses, and wrote the manuscript. This study is part of her research program on complexity and agroecosystems. AE: Conceived, designed and conducted the research, directed the diversity analyses, and wrote the manuscript. This study is part of her research program on microbial diversity and interactions.

## Conflict of Interest Statement

The authors declare that the research was conducted in the absence of any commercial or financial relationships that could be construed as a potential conflict of interest.
