# Supplementary material for: Seasonal Changes in a Maize-Based Polyculture of Central Mexico Reshape the Co-occurrence Networks of Soil Bacterial Communities
Source: Front Microbiol. 2017 Dec 18;8:2478. doi: 10.3389/fmicb.2017.02478 (PMC5741676; doi:10.3389/fmicb.2017.02478)
Supplement: Supplementary file 1 [file Table_1.DOCX]

**Table S1**. Number of samples per analysis per plot.

|  | TIME POINTS | | | | | |
| --- | --- | --- | --- | --- | --- | --- |
| PLOTS | *t1* | | *t2* | | *t3* | |
| **Analyses** | **Physicochemical** | **Sequencing** | **Physicochemical** | **Sequencing** | **Physicochemical** | **Sequencing** |
| F | 5 | 5 | 5 | 10 | 5 | 10 |
| L | 5 | 2 | 5 | 10 | 5 | 9 |
| R | 5 | 5 | 5 | 10 | 5 | 10 |
| T | 5 | 5 | 5 | 9 | 5 | 5 |
| **Total samples** | **20** | **17** | **20** | **39** | **20** | **34** |

**Table S2. PERMANOVA and post-hoc Wilcoxon test for differences on soil physicochemical parameters across plots and sampling times.**

PERMANOVA results of main factors

| Factor | df | SS | MS | F | R^2^ | *P* |
| --- | --- | --- | --- | --- | --- | --- |
| Time | 2 | 0.13943 | 0.069714 | 4.5487 | 0.09385 | **0.005994**** |
| Plot | 3 | 0.52515 | 0.175050 | 11.4215 | 0.35350 | **0.000999***** |
| Time:Plot | 6 | 0.17730 | 0.029559 | 1.9281 | 0.11935 | 0.060939 |
| Residuals | 42 | 0.64371 | 0.015326 | 0.43330 |  |  |
| Total | 53 | 1.48559 | 1.00000 |  |  |  |

Signif. Codes for P values: 0 '***' 0.001 '**' 0.01 '*' 0.05 '.' 0.1 ' ' 1

df=degrees of freedom; SS= sum of squares; MS= mean sum of squares; F=Pseudo-F (value by permutation); *P*=p-value based on 1000 permutations

Wilcoxon rank sum test with continuity correction

| Comparison | pH | Total C | Total N | Total P | C:N ratio | C:P ratio |
| --- | --- | --- | --- | --- | --- | --- |
| *t1* vs *t2* | **1.3e-05***** | 0.3424 | 0.2235 | 0.2814 | 0.1543 | **0.02272*** |
| *t2* vs *t3* | **0.03626*** | 0.6808 | 0.9485 | 0.1989 | 0.8246 | 0.8993 |
| *t1* vs *t3* | 0.06092 | 0.1687 | 0.193 | 0.44 | 0.2611 | **0.02467*** |

Signif. Codes for P values: 0 '***' 0.001 '**' 0.01 '*' 0.05 '.' 0.1 ' ' 1

**Table S3. Indices of the networks of the four plots.** When applicable, ± values correspond to standard deviation.

|  | Sampling time | | | |
| --- | --- | --- | --- | --- |
| Network index | F | L | R | T |
| Number of nodes | 315 | 320 | 314 | 332 |
| Number of edges | 1615 | 1391 | 1838 | 1673 |
| Connectivity | 10.253±7.667 | 8.694±6.623 | 11.707±9.495 | 10.078±7.721 |
| Clustering coefficient | 0.240±0.204 | 0.213±0.201 | 0.219±0.177 | 0.237±0.210 |
| Betweenness centrality | 0.010±0.056 | 0.008±0.009 | 0.011±0.057 | 0.007±0.008 |
| Closeness centrality | 0.334±0.126 | 0.312±0.128 | 0.345±0.158 | 0.319±0.106 |
| Average shortest path length | 3.184±0.691 | 3.368±0.785 | 3.174±0.803 | 3.264±0.692 |
| Network density | 0.033 | 0.027 | 0.037 | 0.030 |
| Network heterogeneity | 0.747 | 0.761 | 0.810 | 0.765 |
| Network centralization | 0.079 | 0.074 | 0.091 | 0.085 |
| Power Law of node degree, R^2^ | 0.643 | 0.690 | 0.694 | 0.695 |
