# Supplementary material for: Seasonal Changes in a Maize-Based Polyculture of Central Mexico Reshape the Co-occurrence Networks of Soil Bacterial Communities
Source: Front Microbiol. 2017 Dec 18;8:2478. doi: 10.3389/fmicb.2017.02478 (PMC5741676; doi:10.3389/fmicb.2017.02478)
Supplement: Supplementary file 3 [file Image_1.PDF]

SUPPLEMENTARY MATERIAL

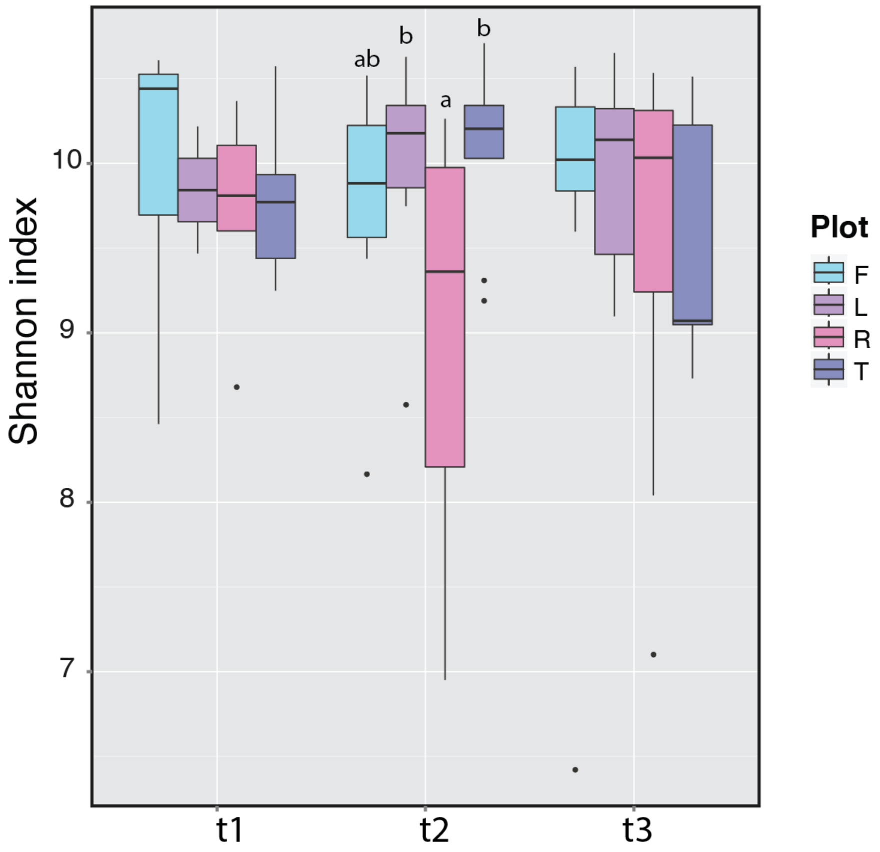

Figure S1. Alpha diversity (Shannon index) through time per plot. Significant differences were found only on *t2* (ANOVA $F(3,35)=3.747$ $p=0.0196$). Letters signify significant differences ($p<0.05$) according to post hoc Tukey test.

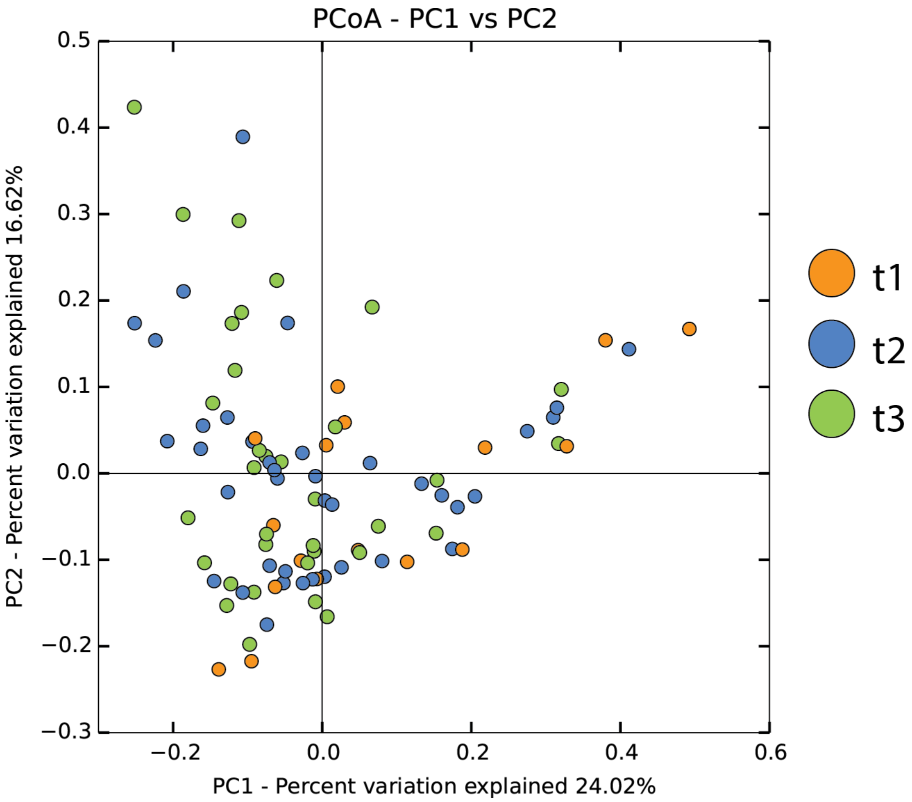

Figure S2. PCoA (PC1 vs PC2) based on weighted unifrac distances (beta diversity). Permutational multivariate analysis of variance using distance matrices (Adonis) showed no significant differences across time (Pseudo-F(2,87)=1.4254 p=0.102).

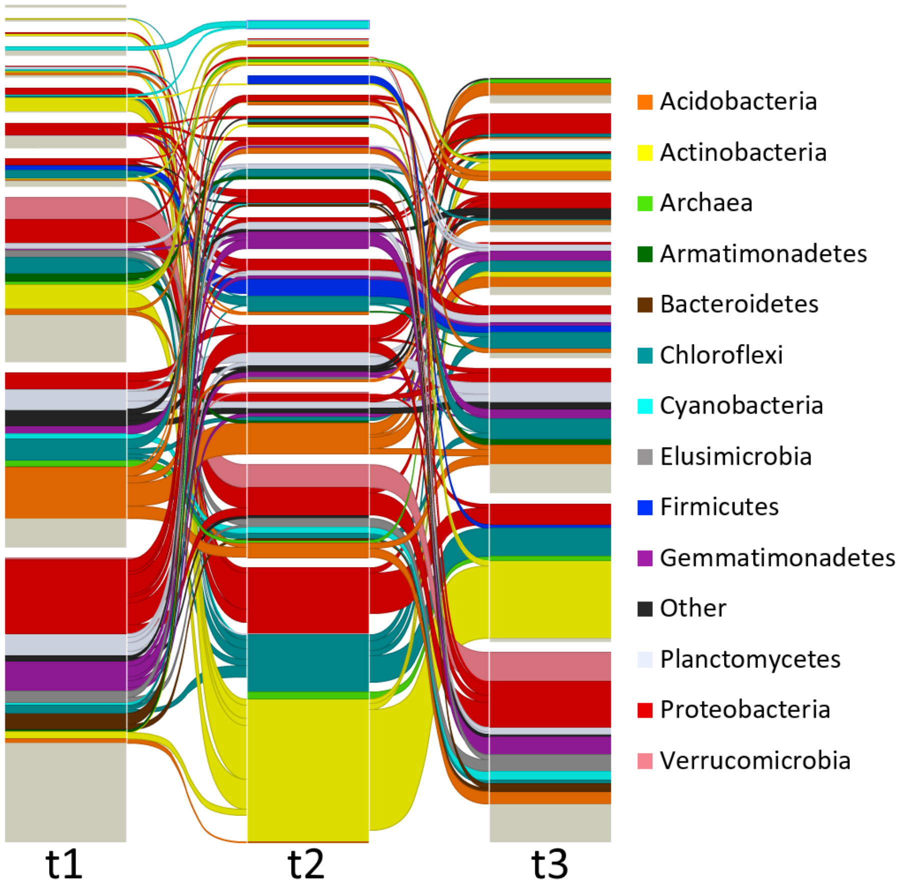

Figure S3. Alluvial diagram of three times. Colors represent taxa for all modules of the early rain season (*t2*). The blocks correspond to modules at three times (*t1*, *t2* and *t3*) and the colors correspond with taxa.

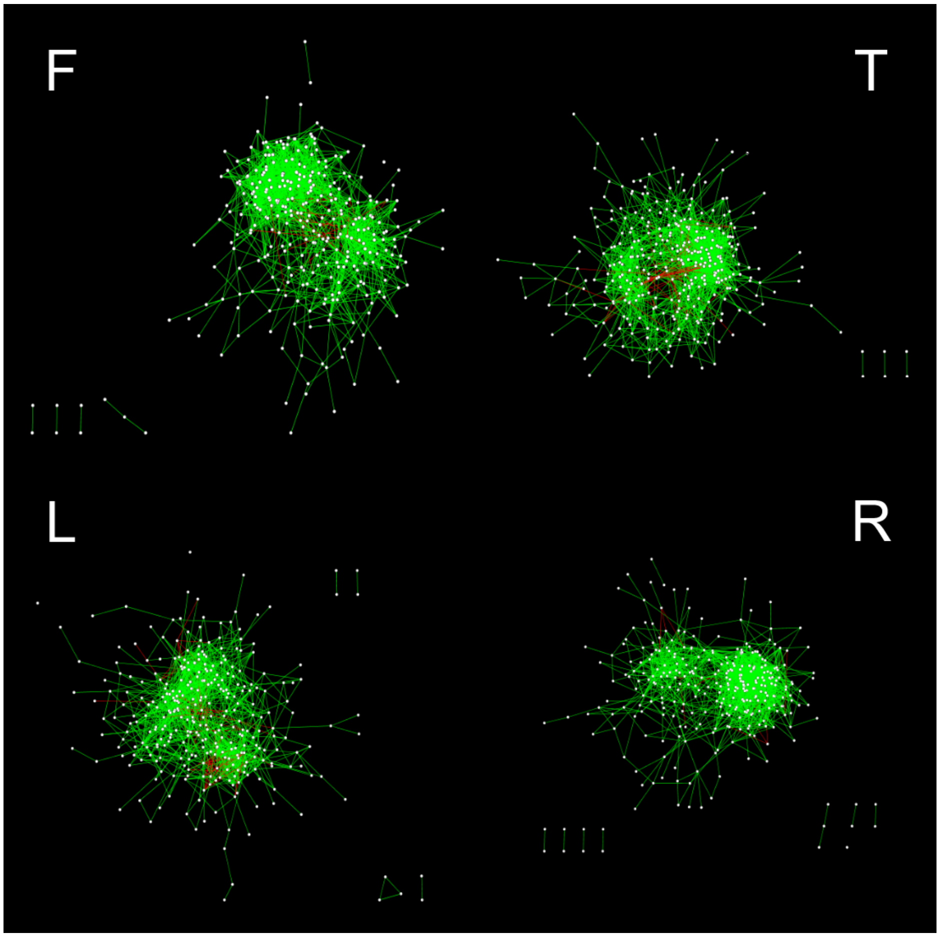

Figure S4. Co-occurrence networks of the four plots. Edges in green indicated co-presence while edges in red indicate mutual exclusion.

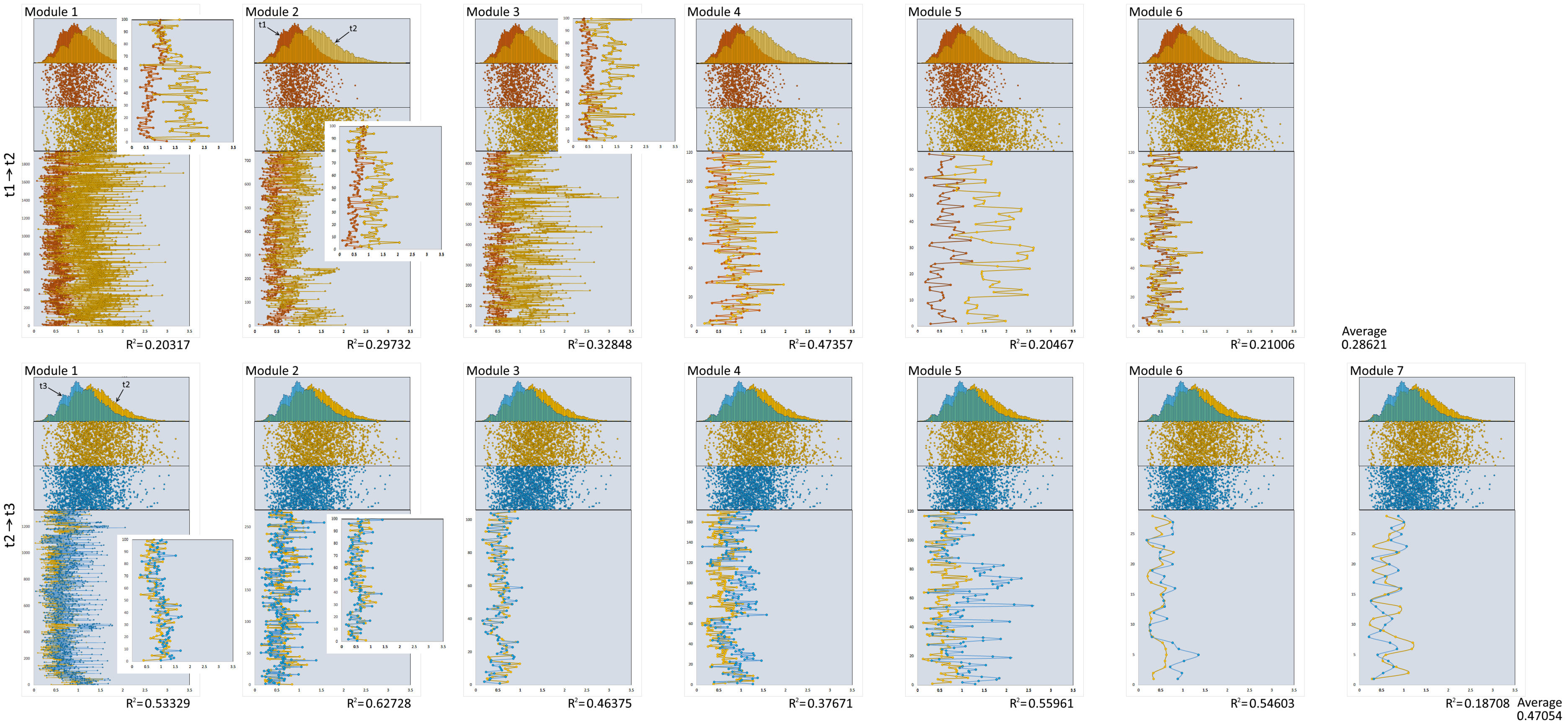

Figure S5. Comparisons between nodes' pairwise distances of time-points for the main modules. The histograms at the top of each panel represent the distribution of the pairwise distances in the entire networks (*t1* and *t2* or *t2* and *t3*); the cloud of points below represent the distances (horizontal axis) of also of the entire networks. The main charts of each panel represent all the pairwise of the respective module with a vertical position that is meaningless, it only corresponds to the order of the pairwise comparisons between nodes (which is the same for both networks). Each panel has two sets of distances and the distances are linked by a line only for visualization purposes. Small charts in some panels are amplified subsets of distances for better visualization. The values below panels represent correlation values.

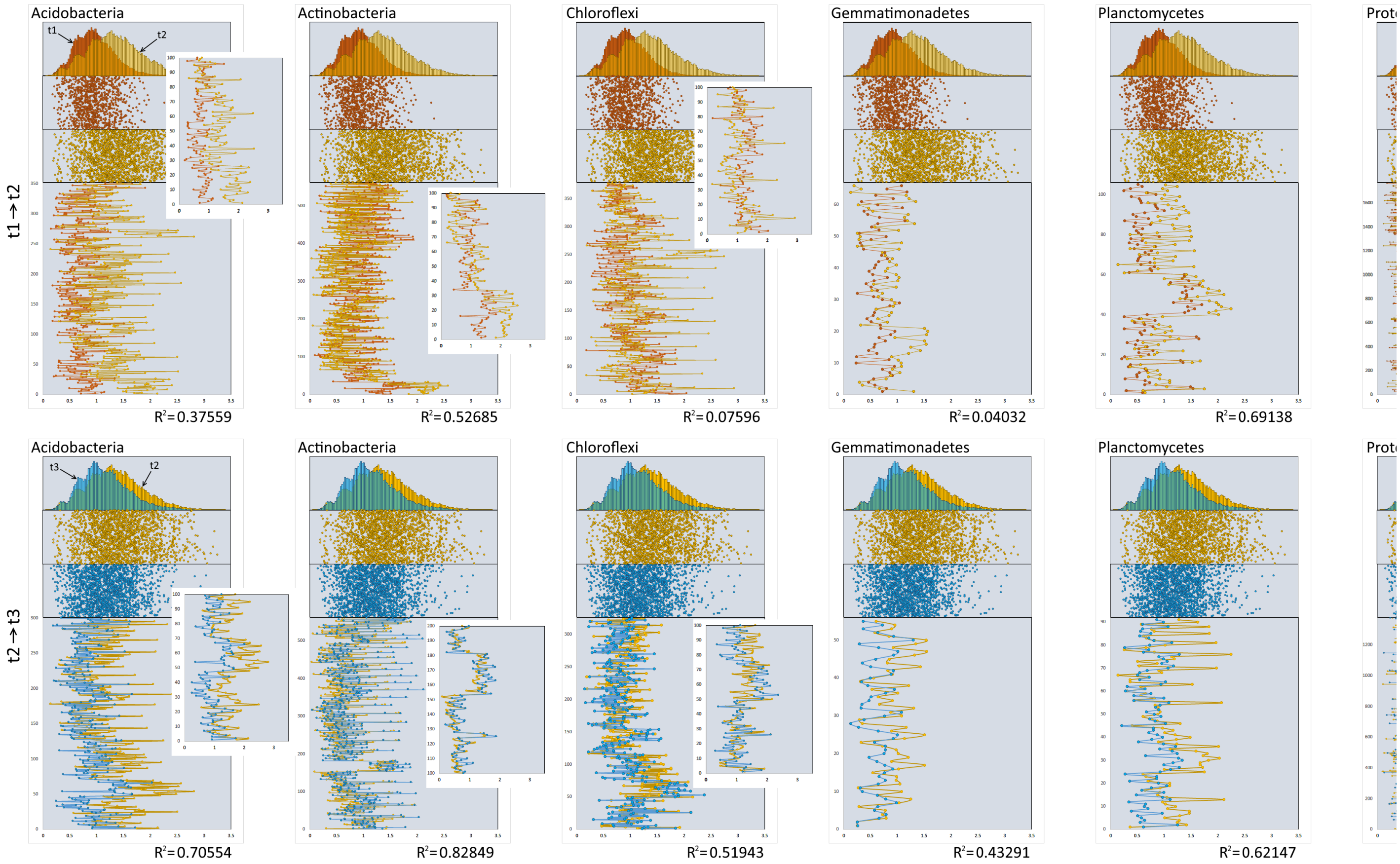

Figure S6. Comparisons between nodes' pairwise distances of time-points for the main taxa. The histograms at the top of each panel represent the distribution of the pairwise distances in the entire networks (*t1* and *t2* or *t2* and *t3*); the cloud of points below represent the distances (horizontal axis) of also of the entire networks. The main charts of each panel represent all the pairwise of the respective taxon with a vertical position that is meaningless, it only corresponds to the order of the pairwise comparisons between nodes (which is the same for both networks). Each panel has two sets of distances and the distances are linked by a line only for visualization purposes. Small charts in some panels are amplified subsets of distances for better visualization. The values below represent correlation values**.**
